# Molecular Mechanisms of GPCR Signaling: A Structural Perspective

**DOI:** 10.3390/ijms18122519

**Published:** 2017-11-24

**Authors:** Vsevolod V. Gurevich, Eugenia V. Gurevich

**Affiliations:** Department of Pharmacology, Vanderbilt University, Nashville, TN 37232, USA; eugenia.gurevich@vanderbilt.edu

**Keywords:** GPCR, G protein, GRK, arrestin, conformational change, cell signaling

## Abstract

G protein-coupled receptors (GPCRs) are cell surface receptors that respond to a wide variety of stimuli, from light, odorants, hormones, and neurotransmitters to proteins and extracellular calcium. GPCRs represent the largest family of signaling proteins targeted by many clinically used drugs. Recent studies shed light on the conformational changes that accompany GPCR activation and the structural state of the receptor necessary for the interactions with the three classes of proteins that preferentially bind active GPCRs, G proteins, G protein-coupled receptor kinases (GRKs), and arrestins. Importantly, structural and biophysical studies also revealed activation-related conformational changes in these three types of signal transducers. Here, we summarize what is already known and point out questions that still need to be answered. Clear understanding of the structural basis of signaling by GPCRs and their interaction partners would pave the way to designing signaling-biased proteins with scientific and therapeutic potential.

## 1. Introduction

G protein-coupled receptors (GPCRs) are the largest family of signaling proteins in animals. Among mammals, elephants hold the record with >3400 GPCR subtypes (Available online: http://sevens.cbrc.jp/). Humans express >800 different GPCRs, which are targeted by a greater number of clinically used drugs than any other protein family. Therefore, the molecular mechanisms of GPCR signaling have attracted close attention for several decades.

Rhodopsin, now considered a prototypical class A GPCR, was cloned before the idea that there is a class of rhodopsin-like receptors appeared, so it was compared to bacteriorhodopsin, a known protein with seven trans-membrane α-helices [1]. The cloning of the β2-adrenergic receptor a few years later revealed a similar topology [2], so 1986 should be considered as a year when the existence of a class of seven trans-membrane domain receptors, now called GPCRs or 7TMRs (seven trans-membrane domain receptors), was first demonstrated. The classification into five main families [3], which appears to hold water even today, brought certain order to the subsequent avalanche of GPCR sequences. 

**Figure 1:**
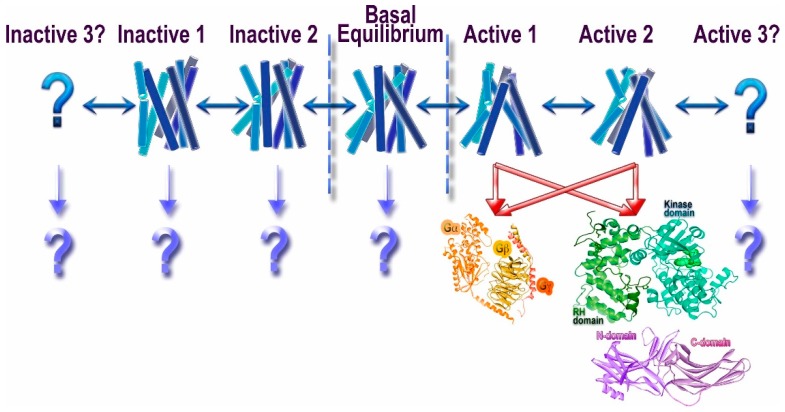
Conformational heterogeneity of G protein-coupled receptors (GPCRs) and signaling. Unliganded GPCRs appear to exist in the equilibrium between multiple conformations (basal equilibrium), which the agonists partially shift towards active conformations [10]. It is likely that there are several “active” conformations of GPCRs (shown as Active 1, 2, and 3). The most realistic scenario is that the great majority of active conformations effectively couple to G proteins, GRKs, and arrestins. However, there likely are some that might preferentially engage distinct signal transducers, such as different G proteins, GRKs, and/or arrestins (this phenomenon is called biased signaling). While the idea that the receptor conformations preferred by G proteins and GRKs/arrestins do not fully overlap appears attractive, we do not have direct structural evidence to support it. Similarly, another idea that phosphates in different positions on the cytoplasmic GPCR elements encode distinct conformations of bound arrestins and therefore functional outcomes [11,12] is enticing, but it also awaits supporting structural evidence. The binding of presumably inactivating inverse agonists also does not shift the equilibrium to a single conformation, suggesting that there might be multiple different inactive states (shown as Inactive 1, 2, and 3). Whether any of these conformations facilitate the engagement of distinct interaction partners remains to be elucidated (shown as “?”).

Rhodopsin also was the first GPCR, for which it was shown that activation involves a rigid body motion of trans-membrane α-helices [4] resulting in opening up of a cavity on the cytoplasmic side of the membrane. By that time, is was shown (again, in the visual system) that several proteins preferentially bind light-activated rhodopsin [5]. Thanks to numerous subsequent studies, today we know that three classes of proteins prefer active GPCRs over inactive: G proteins, G protein-coupled receptor kinases (GRKs), and arrestins (Figure 1). The latter preferentially bind to active phosphorylated receptors [6]. It appears that all three classes engage the cavity between the helices that opens on the cytoplasmic side of GPCRs upon receptor activation [7,8,9].

Thus, proteins belonging to these three families specifically engage activated GPCRs, which makes them candidate signal transducers. Classical view posits that GPCRs signal via G proteins (hence the name), whereas their phosphorylation by GRKs and subsequent arrestin binding serves to desensitize receptors [13]. This model implied that G proteins, GRKs, and arrestins recognize the same active GPCR conformation, in line with the idea that GPCRs exist in two distinct conformations, active and inactive [14]. However, biophysical studies suggested that GPCRs can exist in multiple conformations [10,15], including several distinct active conformations, depending on the ligand (Figure 1). The conformational equilibrium of GPCRs can never be shifted to a single inactive or active state, which likely explains documented constitutive activity of many receptors [10]. This complex behavior of GPCRs opened the possibility that different proteins interacting with activated GPCRs might prefer distinct receptor conformations. Indeed, quite a few ligands of several GPCRs were shown to preferentially activate G protein- or arrestin-mediated signaling, a phenomenon that was termed signaling bias [16] (Figure 1). It should be noted that the evolution apparently created GRKs and arrestins to suppress G protein-mediated signaling, so that in case of native ligands the families of conformations conducive to the binding of G proteins, arrestins, and GRKs largely overlap. However, even minor non-overlap can be exploited therapeutically by designing ligands pushing GPCRs into those rare conformations that are good enough for G protein, but not GRK or arrestin binding, or vice versa. The idea that certain GPCR conformations specifically enhance receptor interactions with some, but not all potential signal transducers appears very attractive. Indeed, synthetic agonists that facilitate G protein signaling but induce minimal desensitization (i.e., do not effectively enhance the binding of GRKs and/or arrestins) have been developed. There are fewer examples of ligands that promote arrestin binding but do not effectively induce G protein coupling. At the moment, we do not know which GPCR conformations favor which interaction partner, and how the structure of agonists needs to be changed to favor G proteins, as opposed to GRKs and/or arrestins. To complicate things further, it is conceivable that preferential engagement of particular signal transducers could be governed by the differences in conformational dynamics and/or kinetic rates between interchanging states rather than defined, distinct conformations. All of this also applies to the selection of a particular G protein in case of numerous GPCRs that couple to more than one subtype, or even to G proteins of different subfamilies.

Thus, from structural perspective, two sets of questions must be asked regarding the mechanisms of GPCR signaling. First, what are the receptor conformations and/or conformational dynamics that G proteins, GRKs, or arrestins prefer? How big is the “loophole” (from the viewpoint of evolution) of non-overlapping states that can be exploited for therapeutic purposes? Second, what structural changes in signal transducers occur upon their binding to active GPCRs? In case of GRKs and arrestins, it is also important to elucidate conformational requirements for their receptor-independent functions [17,18,19]. 

While the ultimate answers are still elusive, several recent biophysical and structural studies addressed these questions. Below, we review the answers gleaned in the last few years, primarily focusing on the phenomena that were not covered in recent reviews and/or allow different interpretations, which were not previously pointed out. Arrestins are described in greater detail because of our own experience. However, we believe that the comparison of the three classes of proteins transducing signals from cell-surface GPCRs to intracellular milieu has an added value. 

## 2. GPCRs

Traditionally, it was assumed that GPCRs exist in two distinct conformations, active and inactive. This notion was the basis for the classical extended ternary complex model of GPCR-driven signaling [14]. This view implied that the active GPCR conformation preferred by G protein, GRKs, and arrestins is one and the same. However, biophysical experiments with purified fluorescently labeled β2-adrenergic receptor (β2AR) showed that the receptor can exist in multiple conformations, and that its conformational equilibrium is affected both by bound ligand and the presence of the cognate G protein [15]. The structure of agonist-liganded β2AR was puzzling: the receptor looked suspiciously similar to its structure with an inverse agonist carazolol [20]. Recent comprehensive biophysical study using both nuclear magnetic resonance (NMR) signals from ^19^F labeled Cys265 at the cytoplasmic end of trans-membrane helix 6 (TM6) and double electron-electron resonance (DEER) distance measurements between nitroxides introduced at position 148 at the cytoplasmic end of TM4 and 266 at the cytoplasmic end of TM6 revealed the complexity of the conformational equilibria of β2AR [10]. This study showed that ligand-free β2AR exists predominantly in two inactive conformations with rapid (hundreds of microseconds) transition between them. Even inverse agonists, while appreciably shifting the equilibrium, do not push the receptor into a single conformation. Interestingly, with bound agonist conformational heterogeneity increases, so that the receptor samples both inactive conformations, an intermediate one, and an active one, with the latter representing only a relatively small fraction of receptor population. The fully active conformation is stabilized only by G protein-mimicking single-chain camelid antibody (nanobody), but even then the heterogeneity persists [10]. These data explain the similarity of β2AR crystal structures with bound inverse agonist and high-affinity agonist. In addition, in agreement with these data, the structure of the same receptor with an agonist and bound nanobody stabilized the receptor in the high agonist affinity state, revealing much greater conformational changes on the cytoplasmic side than with agonist alone [21]. It is important to note that we do not currently have a full description of the conformational space explored by a GPCR under any condition: some states might be indistinguishable by NMR signal from ^19^F labeled Cys265, just like the intermediate and active states revealed by NMR were indistinguishable by the measurements of the distance between positions 148 and 266 using DEER [10]. Thus, any study, however comprehensive, can provide only a limited number of conformational states of the receptor, but not the full spectrum. An attractive idea of therapeutically exploitable biased signaling [22,23,24], implying that GPCRs can have distinct conformations preferred by G proteins, GRKs, and/or arrestins (Figure 1), is still awaiting experimental support.

The structure of the signaling β2AR complex with Gs (stabilized by a different nanobody that binds receptor-associated G protein) revealed an even larger movement of the cytoplasmic end of TM6 and identified receptor and G protein elements involved in their interaction [7]. Unexpectedly, this structure showed that only the Ras domain of the G protein α-subunit directly contacts the receptor [7]. Despite efforts of many labs, this breakthrough study remained the only GPCR-G protein complex structure available for several years. Based on this finding, another group designed a mini-G protein, essentially a mutated Ras domain of Gs α-subunit, and crystallized it with adenosine A2A receptor [25]. This work confirmed key findings of the original β2AR-Gs structure, thereby extending them to another class A receptor, although the use of engineered mini-G protein instead of a natural heterotrimer was a caveat. As crystallization of GPCR-G protein complexes remained tricky, two groups took advantage of improved cryo-electron microscopy (cryo-EM) technology to solve structures of two agonist-liganded class B receptors, calcitonin [26] and glucagon-like peptide-1 (GLP-1) [27], in complex with Gs. Both structures confirmed the previous idea that in class B GPCRs agonist peptides simultaneously bind to the large extracellular N-terminal domain and the pocket between helices occupied in class A GPCRs by their small molecule ligands. Both revealed significant conformational rearrangements of the extracellular part of the seven TM bundle to accommodate bound peptide, as well as a sharp kink in the TM6 necessary to ensure the outward movement of its cytoplasmic end to create a G protein-binding cavity. 

Thus, at the moment, we have one atomic resolution crystal structure of heterotrimeric G protein with class A receptor, β2AR; a crystal structure of engineered mini-G protein with another class A receptor, adenosine A2A; and two lower resolution structures of active class B GPCRs in complex with G protein solved using cryo-EM. All structures show the interaction of the C-terminal α-helix of G protein α-subunit with the cavity that opens on the cytoplasmic side of GPCRs upon receptor activation and large outward movement of the cytoplasmic end of TM6 necessary to create this cavity. The main limitation of the available data is that in all cases the structures contain the same Gs protein (or its engineered mini-version), so that we cannot be sure that other classes of G proteins engage active GPCRs in the same way. Another limitation is that so far only one structure solved by X-ray crystallography contains natural heterotrimeric G protein. We certainly need structures with the highest possible resolution of GPCR complexes with other G proteins to draw general conclusions regarding GPCR-G protein interactions. These structures and/or biophysical studies would also show whether GPCRs of different classes undergo the same conformational changes upon activation. 

Most importantly, we need unambiguous structural evidence for the basis of biased signaling, i.e., demonstration that distinct GPCR conformations are preferentially engaged by different signal transducers: G proteins, GRKs, and arrestins (Figure 1). In case of many GPCRs that couple to more than one kind of G proteins, it is tempting to think that the conformational requirements are also different. Thus far, these interesting ideas are largely speculative.

## 3. Signal Transducers

### 3.1. G Proteins

As the structural basis of GPCR-G protein interaction and GPCR-mediated signaling were extensively reviewed (e.g., see [28] for the latest), here we only focus on the aspects that received less attention and/or pertain to the similarities of the interactions of all three classes of signal transducers, G proteins, GRKs, and arrestins, with active GPCRs. 

The structure of the β2AR-Gs complex [7] revealed one important conformational change induced by the active GPCR in cognate G protein: opening of the cleft between Ras and helical domain of the α-subunit, necessary for the release of the GDP bound to the inactive G protein. The main caveat of this structure was the fact that stabilizing nanobody was bound between these two domains of the G protein α-subunit, pushing them far apart. Additional studies using complementary techniques, hydrogen-deuterium (H/D) exchange [29] and single-particle EM [30] showed that in the nucleotide-free state there is no fixed orientation of the helical domain relative to the Ras domain of the G protein α-subunit: the helical domain is detached from the Ras domain and “dangles” freely.

Distance measurements between introduced spin labels in the Ras and helical domain in free and receptor-bound G protein using double electron-electron resonance (DEER) independently showed large movement of the two domains relative to each other, with some distances changing up to 20 Å [31]. Importantly, in this case distance distributions in receptor-bound G protein were fairly wide, supporting the notion that released helical domain “dangles” and does not have a fixed position in the receptor-bound nucleotide-free form of G protein. This study also showed that when the separation of the two domains was impeded by disulfide cross-linking, nucleotide exchange was severely suppressed, demonstrating that domain separation is necessary for this process [31]. Another important finding of this study was that the C-terminal α5 helix in the Gα subunit transduces the signal from the receptor to the distant nucleotide-binding pocket: the introduction of five helix-breaking Gly residues between the receptor-binding tip and the rest of the α5 helix prevented receptor-induced nucleotide release [31]. Follow-up biochemical and modeling studies revealed further molecular details of the mechanism of signal transduction from the receptor to the nucleotide-binding pocket in the G protein α-subunit [32,33,34].

The fact that receptor-G protein complex has higher agonist affinity than free receptor was discovered by the Lefkowitz group in the late 1970s. In the extended ternary complex model, this phenomenon was interpreted as higher agonist affinity of active, as opposed to inactive, receptor conformation [14]. The study of the binding of various ligands to the β2AR in the presence of Gs or G protein-mimicking nanobody revealed the structural basis of this phenomenon [35]. It was shown that the binding to the receptor of both nucleotide-free G protein and its nanobody mimic pushes ligand-binding site into a more closed conformation, impeding the binding as well as dissociation of all orthosteric ligands, agonists and antagonists [35]. Thus, this study demonstrated that there is the flow of information from the G protein-binding pocket to the ligand-binding site and this is achieved via conformational changes in the GPCR, similar to the flow of information from ligand-binding site to the cytoplasmic surface of GPCRs, where G proteins bind. Thus, the two sites are allosterically connected, as should be expected since agonists promote GPCR signaling to G proteins.

Interestingly, signaling-biased G proteins were designed long before any structural information became available. An early finding that the C-terminus of the α-subunit of visual G protein transducin specifically interacts with light-activated rhodopsin and stabilizes its signaling-competent Metarhodopsin II state [36] was extended to other GPCR-G protein combinations. Based on the discovery that very few C-terminal residues of the α-subunit determine receptor specificity of G proteins [37,38], chimeric G proteins with the bulk representing Gq, which directs the signaling to Ca^2+^ mobilization, equipped with Gα C-terminus that determines receptor specificity derived from other Gα subfamilies, were designed [39]. These chimeras are now widely used in the field, as they allow monitoring easily detectable intracellular Ca^2+^ signal as a readout for activation of any GPCR [39]. The structural basis for this phenomenon was demonstrated later by solving the co-structures of signaling-competent opsin [40] and opsin-derived all-trans-retinal-liganded Metarhodopsin II [41] with a modified peptide representing the C-terminus of transducin Gα. Conceivably, similar G protein engineering can be used to construct chimeric Gα subunits that would link any GPCR to a G protein-activated pathway of choice initiated by other than Gq G protein subfamilies, but this approach still needs to be tested experimentally.

Even though this aspect was not emphasized before, the hybrid Gα subunits studies strongly suggest (but do not prove beyond reasonable doubt) that G proteins of different subfamilies do interact with GPCRs similarly—otherwise, chimeric Gα subunits would not successfully couple various GPCRs to Ca^2+^ signaling. Thus, structural information gleaned from GPCR-Gs complexes likely has predictive value. However, this hypothesis still needs to be tested by solving the structures of complexes of other G proteins with their cognate GPCRs. 

It is also important to keep in mind that while the interactions of GPCR-activated G proteins with classical effectors, such as adenylyl cyclase, phospholipase C, or PDE phosphodiesterase in photoreceptors, are well documented, there is growing evidence for non-canonical signaling of G proteins to the microtubules, actin cytoskeleton, mitochondria, Golgi membranes, and even transcription factors (reviewed in [42]). In some cases, there are intriguing indications that heterotrimeric G proteins can be activated by non-GPCR interaction partners [42]. Molecular mechanisms and the structural basis of this non-canonical activation remain to be elucidated.

### 3.2. GRKs

Unfortunately, structurally GRKs are the least studied proteins among the three families that specifically bind active GPCRs. GRKs belong to the AGC kinase super-family [17] (named for protein kinases A, G, and C discovered earlier). However, unlike the other AGC kinases, such as PKA or PKC, GRKs do not have a consensus phosphorylation site, but appear to phosphorylate various serines and threonines in the C-terminus and other cytoplasmic GPCR elements regardless of their sequence context. However, GRKs of the GRK2/3 subfamily apparently phosphorylate different serines and threonines than ubiquitous members of GRK4 subfamily, GRK5/6, as was directly demonstrated with some GPCRs [12,43] or deduced from different functional consequences of this phosphorylation in case of other receptors [44,45]. In contrast to PKA, PKC, and CaMK, no consensus phosphorylation sequence for any GRK has been identified so far. Considering that the four ubiquitously expressed GRK subtypes, GRK2/3/5/6 [17], apparently phosphorylate hundreds of different GPCRs with limited sequence homology, it is highly unlikely that such a consensus exists. It is tempting to speculate that the position of particular serine and threonine residues in the C-termini or cytoplasmic loops of the receptor in the GPCR-GRK complex determines which ones are targeted. This idea implies that different GPCR-associated GRKs have distinct poses in the complex. High-resolution structures of several GRKs in complex with the same GPCR, such as β2-adrenergic receptor where distinct targets of GRK2 and GRK6 were identified [12], are needed to test this model.

In the basal (inactive) state of AGC kinases, the two lobes of their kinase domain are misaligned, and their proper alignment accompanies kinase activation. Another peculiarity of GRKs, as opposed to the other members of the AGC family, is that they are not activated by small molecules or the phosphorylation of the activation loop, but by physical interaction with activated GPCRs [46], whereupon they can phosphorylate anything available to them, even exogenous peptides [47]. While this fact was established a long time ago, the exact mechanism of GRK activation remained elusive: in the basal state, the lobes of their kinase domain are misaligned, like in other AGC kinases, but it was unclear how receptor binding pushes them into alignment. The first clues came from the structure of GRK6, where this kinase formed a dimer [48]. In this case, the lobes were found to be closer to alignment expected in the active AGC kinase than in all previous GRK structures. It appeared that the two molecules in a dimer serve as surrogate activators of each other, representing the first case of AGC kinase activation via protein-protein interactions. 

As GRKs only transiently interact with GPCRs, there are no crystal structures of the complex. Recently, the first structure of GPCR-bound GRK, deduced from biochemical studies assisted by molecular dynamics and computational docking, was proposed [9]. This study used β2AR and GRK5. It revealed large conformational changes between RGS homology (RH) and catalytic domains of GRK5, both of which appear to participate in the receptor binding. Receptor binding-induced conformational rearrangement in the GRK possibly leads to the closure of the two lobes of the catalytic domain necessary for the kinase activity [9]. Another model, based on biochemical assays, H/D exchange, and negative stain EM, was also proposed [49]. Here the authors used pre-activated rhodopsin mutant with GRK1 (also known as rhodopsin kinase) and gain-of-function Q41L mutant of GRK5 and came to the conclusion that the RH domain is the key receptor-binding element of GRKs, although the N-lobe of the kinase domain might also participate in the interaction [49]. Neither model is a true structure, but the fact that they are fairly similar inspires some confidence. Better resolution structures of the complex would be welcome, although this might involve trapping this protein-protein interaction, which nature intended to make very transient, by artificial means. 

In addition to active GPCRs, GRKs phosphorylate numerous other proteins (reviewed in [17]). It is tempting to speculate that GRKs activated by their association with an active GPCR acquire the ability to phosphorylate various non-receptor substrates (as was shown with exogenous peptides [47]), but this model must be tested in the cellular context, which is not a trivial task. Alternatively, GRK interactions with non-GPCR protein partners might also result in the alignment of the two lobes of the catalytic domain, leading to kinase activation. This model can be tested in a simpler in vitro system, although the search for possible non-GPCR activators of GRKs is also a challenge. The third possibility is that well documented basal activity of GRKs is sufficient to act on their non-GPCR substrates, quite a few of which have already been identified (reviewed in [17]). Importantly, only in the first model GRKs act as true transducers of signals from GPCRs, whereas the latter two only imply multi-functionality of these enzymes. 

### 3.3. Arrestins

#### 3.3.1. Receptor Binding-Induced Structural Changes in Arrestins

All four vertebrate arrestins in their basal state have very similar conformations: an elongated two-domain molecule with a “wingspan” of ~75Å The orientation of the two domains relative to each other is maintained by two intra-molecular interactions: the polar core, a network of five interacting virtually solvent-excluded charged residues between the domains, and the anchoring of the C-terminus to the N-domain via hydrophobic side chains [50,51,52,53]. The two domains are vaguely homologous, each being a “sandwich” consisting of seven β-strands with connecting loops. High Arrhenius activation energy of the visual arrestin-1 binding to rhodopsin, suggesting that in the process of receptor binding arrestin undergoes a global conformational change, was reported in 1989 [54]. Early studies (before structural information became available) suggested that receptor binding induces the release of the arrestin C-terminus, which becomes more accessible to proteases [55]. The first systematic structure-function study suggested that arrestins have two separate sites, one responsible for the binding of receptor-attached phosphates, while the other interacting with the parts of a GPCR that change conformation upon activation [56]. However, the binding of visual arrestin-1 to active phosphorylated rhodopsin was many times greater than its binding to inactive phosphorylated or active unphosphorylated states, supporting the notion that the binding must be accompanied by a major conformational rearrangement of the molecule that brings additional elements in contact with the receptor when the latter is activated and phosphorylated at the same time [56]. The deletion of the arrestin-1 C-terminus enhanced the binding to non-preferred forms of rhodopsin, but did not increase it to the level of binding to active phosphorhodopsin, suggesting that additional conformational rearrangements accompanied arrestin activation [56]. Various models of these conformational changes were proposed [6,57], but the experimental evidence was missing at the time.

**Figure 2:**
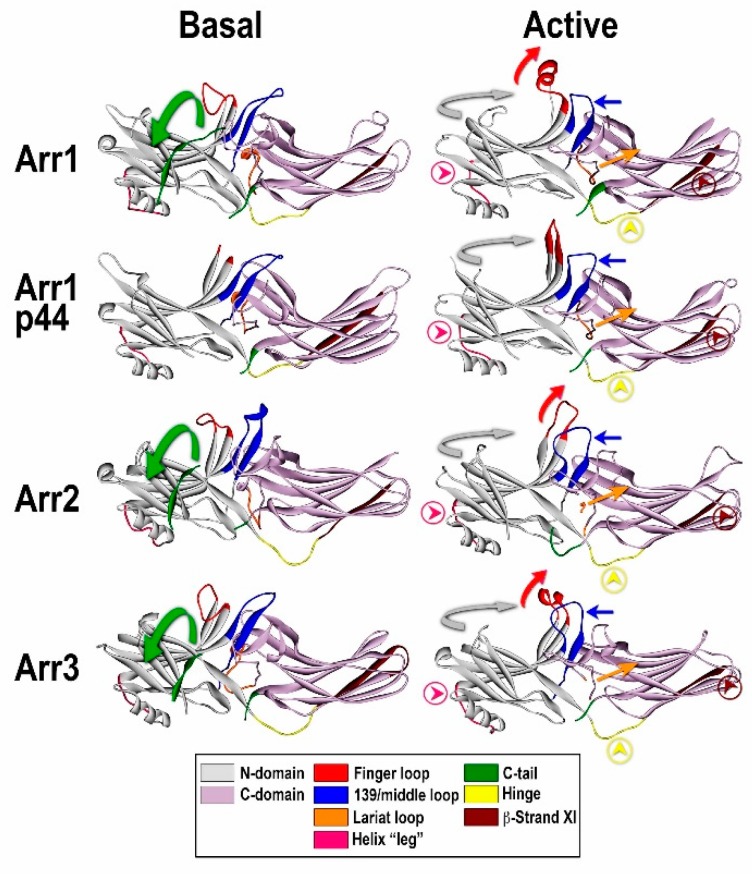
Comparison of the basal and “active” conformations of arrestin proteins. Crystal structures of arrestin-1 (Arr1) (PDB ID 1CF1) (**left**) [51] and 4ZWJ (**right**) [8], arrestin-2 (Arr2) (PDB ID 1G4M) (**left**) [50] and 4JQI (**right**) [63], short splice variant of arrestin-1, p44 (PDB ID 3UGU) (**left**) [65] and 4J2Q (**right**) [66], and arrestin-3 (PDB ID 3P2D) (**left**) [53] and (PDB ID 5TV1) (**right**) [67]. The elements with different conformations in active and inactive arrestins are shown, as follows: the C-tail, green (moves out of its basal position in the cavity of the N-domain in the process of receptor binding; absent in p44); inter-domain hinge, light blue (has essentially the same conformation in all activated arrestin structures, in contrast to a wide range of conformations in the basal state); β-strand XI, dark blue (it is register shifted by one residue in arrestin-2 and -3, which “flips” it by 180 degrees, and by two residues in arrestin-1 and p44, which moves it relative to the other parts of the molecule but does not change the exposed side chains); finger loop, red (moves towards the receptor, the tip becomes α-helical); 139-loop (also known as middle loop), violet (moves towards the N-domain); lariat loop extension, magenta (moves lariat loop towards the N-domain, out of its basal position, which removes two out of three negative charges from the polar core); the connector of the α-helix in the N-domain, pink. The twist of the two domains relative to each other (N-domain, teal; C-domain, gray) by 17–20 degrees is indicated by arrows.

The first study of the spin-labeled arrestin-1 confirmed the release of the C-terminus: relatively short distance between this part of the molecule and the N-domain, which matched crystal structure, became a wide distribution of much longer distances upon rhodopsin binding [58], suggesting that in the complex with the receptor released arrestin C-terminus “flops around” and does not have a fixed position. Interestingly, in both non-visual subtypes, arrestin-2 and -3, receptor binding was also found to induce the release of the C-terminus, but in these arrestins the C-termini of the receptor-bound forms appeared to have several preferred positions [59]. NMR study of arrestin-1 binding to rhodopsin also confirmed the release of the C-terminus, which was found to be very mobile in free arrestin and became even more mobile in the complex [60]. An additional important finding was that the rest of the molecule, which was flexible in free state, remained very flexible in the complex [60], supporting the idea that there are multiple “active” conformations of the receptor-bound arrestin [61]. A fairly comprehensive study of receptor binding-induced conformational changes in arrestin-1 used the measurements of inter-spin distances in free and rhodopsin-bound arrestin-1 by DEER. A total of 25 distances in doubly spin-labeled arrestin-1 included three classes: 12 inter-domain, eight within the N-domain, and five within the C-domain [62]. The measurements showed that the N- and C-domains did not move much relative to each other, contrary to expectations of a “clam-shell” model that posited that the two arrestin domains grip the cytoplasmic side of the receptor like a pincer [6]. The study showed that a loop earlier implicated in P-Rh* binding (the “finger loop” connecting β-strands V and VI in the N-domain, residues 67–79 in bovine arrestin-1; Figure 2) moves toward the expected position of rhodopsin, but does not change conformation all the way to a fully extended one. A large rather unexpected movement of a neighboring loop in the central crest of the receptor-binding side of arrestin, which contains residue 139 in bovine arrestin-1, away from the adjacent finger loop and towards the N-domain, was also detected. The movement of the “139-loop” (termed middle loop in arrestin-2 [63]) apparently facilitates receptor binding [62,64]. Smaller movements of loops containing residues 157 and 344 at the distal tips of the N- and C-domains [62], corresponding to “plastic” regions of arrestin-1 (defined as elements that have different conformations in the four monomers of the crystal tetramer [51]), were also detected. Remarkably, the loops containing residues 139, 157, and 344 appear to be highly flexible in both free arrestin-1 and the P-Rh* complex [62], which agrees with the findings of the NMR study [60]. 

While these findings shed new light on the receptor binding-induced conformational changes in arrestins, the data did not explain why progressive shortening of the inter-domain hinge (Figure 2) inhibits receptor binding of arrestin-1 [68], as well as non-visual arrestin-2 and -3 (also known as β-arrestin-1 and -2) [69]. Crystal structures of “active” presumably receptor-bound-like C-terminally truncated arrestin-2 in complex with multi-phosphorylated peptide representing the C-terminus of vasopressin 2 receptor [63] and of p44, a short “pre-activated” splice variant of arrestin-1 [66], explained these findings, suggesting that receptor binding is accompanied by ~20 degree rotation of the two arrestin domains relative to each other. Interestingly, domain rotation in receptor-bound arrestins was earlier predicted to require an extended inter-domain hinge by molecular modeling [57]. Both structures also confirmed many of the conformational rearrangements suggested by the DEER study [62]. However, neither of these structures included a bound GPCR. 

The first structure containing both the receptor, which was a chimera with the trans-membrane part of β2-adrenergic receptor and the C-terminus of V2 vasopressin receptor, and arrestin was visualized by a relatively low resolution cryo-EM [70]. Receptor-bound arrestin in this study was found to be in two alternative poses: hanging, attached solely via the phosphorylated receptor C-terminus, and fully engaged, with the center of the arrestin molecule roughly corresponding to the center of the receptor and the long axis of arrestin nearly parallel to the membrane surface [70]. These data were in remarkable agreement with the earlier hypothesis that arrestin has two receptor-binding sites, one engaging receptor-attached phosphates and the other recognizing the active state of the receptor [56]. The interaction solely with the receptor-attached phosphates appeared to match the “hanging” pose, whereas simultaneous binding to the inter-helical cavity, that opens upon GPCR activation [4], and to the phosphates appeared to match the fully engaged pose. Finally, a higher resolution X-ray structure of the arrestin-1-rhodopsin complex has been solved in 2015 [8]. It revealed an inter-domain rotation, movements of the arrestin loops, as well as α-helical conformation of the finger loop in the complex, which was proposed earlier based on the co-structure of the finger loop peptide bound to rhodopsin [71]. Importantly, this structure showed an asymmetric pose of arrestin on the receptor, with the distal tip of the C-domain localized near the expected position of the membrane [8], in agreement with subsequent finding that this part of arrestin-1 actually engages the membrane lipids [72]. The main caveat of this groundbreaking structure was the absence of the phosphorylated rhodopsin C-terminus, which was known to be necessary for the high-affinity arrestin-1 binding to rhodopsin [56]. Further refinement of the arrestin-1-rhodopsin structure with additional data and more advanced software improved resolution and allowed the localization of the phosphorylated rhodopsin C-terminus in the complex, which doubled the measured area of the arrestin-rhodopsin contact [73]. In addition, this improved structure identified three positive patches on arrestin-1 for the binding of the receptor-attached phosphates and/or negatively charged side chains of the receptor residues. The number of these patches was in striking agreement with previous in vitro [74] and in vivo [75] findings that three rhodopsin-attached phosphates are necessary for high-affinity binding of arrestin-1 that ensures rapid shutoff of rod photoreceptor response to a single photon. The identification of three positive phosphate-binding patches on arrestin allowed the formulation of general rules regarding phosphorylation codes that the receptor needs to have for high affinity arrestin binding [73]. The presence or absence of full or partial codes in different GPCRs was found to correlate with the strength of arrestin interactions, and the conversion of the partial codes into full ones by mutagenesis enhanced arrestin binding to β2AR that engages arrestins only transiently [73].

To summarize, at the moment, we can be fairly sure about several structural features of the receptor-bound arrestins (Figure 2). First, arrestins engage receptor-attached phosphates via positive charges in the N-domain, as was earlier proposed based on the effect of mutations eliminating or reversing the charge of several positively charged side chains in the N-domain [76,77,78]. Second, arrestins engage the cavity that opens between the helices of the active GPCRs via the finger loop, which was also implicated in receptor binding by the previous electron paramagnetic resonance (EPR) [58,79] and structural [71] studies. Third, several additional arrestin elements come into direct contact with the non-phosphorylated parts of the receptor. Fourth, the arrestin C-terminus is displaced from the cavity of the N-domain, being replaced by the phosphorylated receptor C-terminus. Presumably in the case of receptors carrying phosphorylation sites in other elements, such as the third cytoplasmic loop (e.g., M2 muscarinic receptor [80,81]), these parts occupy the same position as the C-terminus of receptors carrying phosphorylation sites in that element. Fifth, the two arrestin domains twist relative to each other by ~17–20 degrees upon receptor binding. It is entirely possible that receptor-independent arrestin activation results in similar conformational rearrangements as the binding to active phosphorylated receptors [82].

#### 3.3.2. Receptor Specificity of Arrestins

Mammalian species express 800–3400 different GPCRs, but only four arrestin proteins. Two of these, arrestin-1 and arrestin-4, are specialized visual, expressed in photoreceptor cells, whereas the other two, arrestin-2 and arrestin-3 (also known as β-arrestin-1 and β-arrestin-2, respectively), serve hundreds of different GPCRs [61]. It is still unclear why there are so few non-visual arrestins, or if they are promiscuous, why do mammals have two, whereas in insects only one promiscuous non-visual arrestin, kurtz, successfully fulfils the same function [18]. Numerous functional differences between the two mammalian non-visual subtypes that are not related to receptor binding have been reported (reviewed in [61]). The comparison of the crystal structures of arrestin-2 [50,83] and arrestin-3 [53] revealed that the C-domain of the latter has one loose β-strand on the receptor-binding surface, which appears to underlie even lower receptor specificity of this subtype, as compared to arrestin-2 [53]. 

Comprehensive mutagenesis of every residue in the arrestin-1 revealed numerous elements affecting receptor binding, as well as an unexpected fact that there are arrestin-1 residues that differentially affect the binding to phosphorylated opsin that does or does not have bound agonist, all-trans-retinal [84], supporting the idea that receptor-bound arrestin can be in different “poses” [70] and conformations [61]. Mapping of the receptor footprint on arrestin using site-directed spin labeling/EPR [58,79], chimeras between rhodopsin-specific arrestin-1 and more promiscuous arrestin-2 [85], and site-directed mutagenesis [79] revealed that receptors engage a fairly large part of the concave sides of the two arrestin domains. However, relatively few residues within this extensive surface significantly affect its receptor preference and drive the binding to the non-phosphorylated parts of the receptor [79]. As fairly high specificity for visual pigments of arrestin-1 showed that receptor-specific arrestins are possible, several studies tested the feasibility of enhancing receptor specificity of non-visual arrestins. This was not a purely academic exercise: many gain-of-function GPCR mutants cause various diseases [86,87], and an enhanced form of arrestin-1 was shown to suppress excessive rhodopsin signaling, which made this compensational strategy potentially feasible as a therapeutic approach [88]. However, virtually every cell expresses 5–25 different GPCRs, so that an enhanced promiscuous non-visual arrestin would simultaneously suppress faulty signaling of overactive GPCR mutant and all the normal GPCRs in the same cell, likely causing unwanted side effects. Thus, therapeutic targeting of mutant overactive GPCRs with an enhanced arrestin requires high receptor specificity of the latter. It was shown that manipulation of a few receptor-discriminator residues can significantly change receptor specificity of arrestin mutants, whereas double mutations yielded up to 50–60-fold preference for certain GPCRs over others [89]. However, the first sets of mutations tested significantly affected the binding to some subtypes, such as β2-adrenergic, M2 muscarinic, D2 dopamine, and Y2 neuropeptide Y receptors, but did not affect the binding to others, such as D1 dopamine and Y1 neuropeptide Y receptors [89,90]. Solved crystal structure of the arrestin-1-rhodopsin complex [8] identified additional receptor-binding arrestin elements that were not targeted before. Indeed, mutagenesis within those elements yielded novel forms of arrestin that discriminated between receptors that were not affected by earlier mutations, as well as between active and inactive states of some GPCRs [91]. These data proved the feasibility of this approach, but indicated that a wider net must be used to construct arrestins specifically targeting intended GPCRs.

#### 3.3.3. Arrestin-Mediated Signaling

As GPCR-dependent arrestin-mediated signaling was recently covered in very comprehensive reviews [61,92,93], here we will focus on several examples of the rather unexpected GPCR-independent modes of arrestin signaling. 

Among the four arrestin subtypes, only arrestin-3 facilitates JNK3 activation via the ASK1-MKK4/7-JNK3 cascade [94,95,96]. This function of arrestin-3 does not depend on its ability to bind receptors, as even receptor binding-deficient mutants facilitate JNK3 activation in cells [95,97]. One of the mutant forms of arrestin-3 was found to bind JNK3 and upstream kinases, but failed to promote JNK3 activation [97]. Importantly, this mutant was shown to act as a dominant-negative “silent scaffold”, recruiting the kinases away from productive scaffolds, thereby suppressing JNK3 activation in a dose-dependent manner [97]. This property made the mutant a potentially useful molecular tool for the suppression of often pro-apoptotic JNK3 activity, which might come in handy in cases where excessive cell death must be prevented, e.g., in degenerative disorders, such as Alzheimer’s or Parkinson’s disease. 

Another cytoprotective tool with similar therapeutic potential, this time based on a different non-visual subtype, arrestin-2, emerged from the unexpected discovery of its role in the core mechanism of programmed cell death [98]. This study showed that caspase cleavage of arrestin-2 generates 1–380 fragment that cooperates with caspase-generated tBid to facilitate the release of cytochrome C from mitochondria, which is the point of no return in the apoptosis of vertebrate cells [99]. The expression of caspase-resistant form of arrestin-2, where the aspartates targeted by caspases were replaced with similarly negatively charged glutamates, which appears to retain other functions of the parental wild type protein, was shown to be cytoprotective. 

The study of the arrestin-3 interaction with JNK3 identified three arrestin-3 elements involved in JNK3 binding, with the N-terminal 25-residue T1A peptide demonstrating the highest affinity for JNK3 [100]. Interestingly, the same short T1A peptide was found to interact with the upstream kinases and facilitate JNK3 activation in cells [101]. In addition to being the smallest MAP kinase scaffold known to date, this peptide, devoid of other arrestin-3 functions, appears to be a potentially useful tool for specific activation of pro-apoptotic signaling, which might provide novel therapeutic options in disorders associated with excessive cell proliferation, such as cancer. However, its ability to reduce cell growth and proliferation still needs to be tested. 

Another unexpected finding was the elucidation of the role of arrestins in cell spreading and motility [102]. It was found that both non-visual subtypes play an important role in focal adhesion dynamics, recruiting clathrin to the microtubules that target focal adhesions, which apparently facilitates focal adhesion disassembly by promoting the internalization of integrins [102]. In the absence of both non-visual arrestins cells have too many abnormally long-lived focal adhesions. As the result, cells spread excessively and have trouble moving [102]. Interestingly, this arrestin function was also shown to be GPCR-independent. 

Thus, arrestins not only mediate numerous signaling pathways initiated by activated GPCRs, but also participate in many receptor-independent signaling processes in the cell, affecting core cellular functions, such as adhesion, motility, survival, and apoptotic death. The structural basis of arrestin interactions with various non-receptor partners involved in GPCR-dependent and—independent arrestin signaling remains to be elucidated.

## 4. Conclusions

Recent structural and biophysical data revealed the mechanisms of GPCR interactions with the three classes of proteins that preferentially bind active receptors, and therefore are likely transducers of GPCR signaling: G proteins, GRKs, and arrestins. The structural studies yielded atomic resolution of G protein and arrestin complexes with GPCRs, whereas the structure of the GPCR-GRK complex was solved by a lower-resolution cryo-EM. Considering that mammals express hundreds of GPCR subtypes, 20 different Gα subunits (that likely form even greater variety of heterotrimeric G proteins with distinct βγ-combinations), and seven GRKs, the information obtained so far, however exciting, is very limited. The field needs the structures of many more receptors in complex with G protein subtypes other than Gs, with non-visual arrestins present in virtually every cell, and with GRKs representing all three subfamilies. Moreover, the structures of active GTP-liganded G protein α-subunits, βγ-subunits, arrestins, and GRKs with a variety of non-receptor partners, as well as biophysical studies of these interactions, are needed. This structural and dynamic information will shed additional light on the molecular mechanisms of cellular signaling and pave the way to the design of signaling-biased GPCRs, G proteins, GRKs, and arrestins for research and therapeutic purposes.
